# Detrusor Myocyte Autophagy Protects the Bladder Function via Inhibiting the Inflammation in Cyclophosphamide-Induced Cystitis in Rats

**DOI:** 10.1371/journal.pone.0122597

**Published:** 2015-04-01

**Authors:** Jiang Zhao, Qixiang Song, Liang Wang, Xingyou Dong, Xingliang Yang, Xinyu Bai, Bo Song, Margot Damaser, Longkun Li

**Affiliations:** 1 Department of Urology, Second Affiliated Hospital, Third Military Medical University, Chongqing, China; 2 Department of Biomedical Engineering, the Cleveland Clinic, Cleveland, OH, United States of America; IISER-TVM, INDIA

## Abstract

Autophagy, a highly conserved homeostatic cellular process that removes and recycles damaged proteins and organelles in response to cellular stress, is believed to play a crucial role in the immune response and inflammation. The role of autophagy in bladder cystitis, however, has not well been clarified. Here we investigate the role of detrusor myocytes autophagy (DMA) in cyclophosphamide-induced cystitis animal model. 164 female Sprague-Dawley rats were randomized into three experimental groups and compared to three control groups, respectively. The expressions of microtubule-associated protein 1 light chain 3 (LC3), p-p70s6k (the phosphorylated form of ribosomal protein S6), SOD2 (superoxide dismutase 2) in the bladder muscular layer were measured using western blot. The co-location of LC3, alpha-smooth muscle actin (α-SMA), and autophagic vacuoles were investigated with double-labeled immunofluorescence and transmission electron microscopy (TEM). The expression of lL-1β, IL-6, IL-8, malondialdehyde (MDA), and glutathione (GSH) in the detrusor layer were analyzed using ELISA. The bladder inflammation and the number of mast cells in the muscular layer were analyzed by histology. The bladder function was evaluated using cystometry. In cyclophosphamide-induced cystitis, autophagy was detected in detrusor myocytes by increased LC3, p-p70s6k expression, and autophagosomes. However, the presence of enhanced inflammation and oxidative stress in the cyclophosphamide-treated group suggest autophagy of detrusor myocytes may not be sufficiently activated. Inflammation and oxidative stress were significantly decreased and the bladder histology and micturition function were significantly improved with rapamycin (RAPA, autophagy agonist) pre-treatment. In contrast, inflammation and oxidative stress were dramatically increased and the bladder histology and function were negatively affected with chloroquine (CQ, autophagy blocker) pre-treated. These findings preferentially provide evidence of the association between DMA and cyclophosphamide-induced cystitis in rats. The autophagy agonist RAPA significantly decreased the inflammation and protected the bladder function, which might be considered as a potential treatment for interstitial cystitis.

## Introduction

Bladder pain syndrome/interstitial cystitis (BPS/IC) is a urological problem characterized by an increase in urinary frequency, urgency, pelvic pain, and other discomforts [1]. BPS/IC is represented by the reduce of the quality of life for 3.3–7.9 million women in the United States [2, 3]. Though there are a number of potential pathogeneses, including infection, autoimmune disorders, toxic substances in the urine, urothelial dysfunction, and neurogenic inflammation, the exact pathogenic mechanisms of BPS/IC have not been well clarified [4, 5]. Immunologic derangement and inflammation play an irreplaceable role in the pathogenesis of BPS/IC [4–6]. Recently, autophagic regulation of inflammation and immunity has been extensively studied [7]. So far, whether autophagy of detrusor myocytes has been involved in bladder inflammatory disorders remains unknown.

Macroautophagy (hereafter referred to as autophagy) plays a housekeeping role by isolating intracellular organelles and protein aggregates, and delivering them to lysosomes for clearance [7]. Increasing evidence suggests that autophagy meticulously orchestrates immune and inflammatory responses. In addition, autophagy contributes to the pathogenesis and progression of a variety of human inflammatory diseases and autoimmune diseases, including Crohn’s disease, liver disease, acute pancreatitis, and intestinal inflammation [7–9]. During an immune response, autophagy demonstrated a protective effect through regulation of the inflammatory transcriptional response [7, 10], negative regulation of inflammasome activation [6, 11], elimination of damaged mitochondria, reduction of reactive oxygen species (ROS) [7, 12], regulation of endoplasmic reticulum (ER) stress, and clearance of apoptotic cells [7, 9].

Autophagy can occur in smooth muscle, like blood vessels, the respiratory tract, and the corpus cavernosum [13–15], suggesting an important role in tissue protection. Little is known about the role and function of autophagy in detrusor myocytes during the pathogenesis of BPS/IC. Cyclophosphamide (CYP), a chemotherapeutic drug, which is effective in the treatment of neoplastic diseases, has been used to induce cystitis in rodents through its toxic metabolite, acrolein, and has been suggested to cause urinary bladder inflammation and urodynamic changes [5]. The CYP-induced cystitis animal model has been commonly used to investigate the effects of an inflammatory response on urinary bladder dysfunction and hyperalgesia in BPS/IC [5, 16–18]. This study aims to investigate the existence and efficacy of autophagy in detrusor myocytes in a cyclophosphamide-induced cystitis animal model.

## Materials and Methods

### Development of a cyclophosphamide-induced cystitis animal model and experimental design

This study was approved by the Research Council and Animal Care and Use Committee of the Third Military Medical University, China (approval no. SYXK 20070002). One hundred and sixty-four female Sprague-Dawley rats weighing 200–230 g were used in this study. One hundred and forty-four of them were randomized into three experimental groups (36 rats each): a cyclophosphamide treated (CYP) group, a CYP+rapamycin-treated (CYP+RAPA) group, and a cyclophosphamide+chloroquine-treated (CYP+CQ) group; and three control groups (12 rats each): a sham CYP-treated control group, a RAPA-treated group, and a CQ-treated group, for western blot, immunofluorescence, electron microscopy and ELISA analysis. The remaining 20 rats were assigned randomly and equally (5 rats each) into 4 groups: a CYP group, a CYP+RAPA group, a CYP+CQ group, and a sham CYP group for cystometry assessment.

The cystitis animal model was developed via a one-time intraperitoneal (i.p.) injection of CYP, 150 mg/kg (Sigma, C0768) mix with 0.6 ml saline as previously described [5]. The sham CYP group received a saline injection only (0.6 ml per rat, i.p.). In the CYP+RAPA or CYP+CQ groups, prior to the injection of the same dosage of CYP to establish the cystitis animal model, rats were pre-treated with either i.p. injection of RAPA, 1 mg/kg (Ruibio, LR7015) [19, 20] or CQ, 60 mg/kg (Sigma, C6628) [21, 22]mixed with 0.4 ml saline once daily for three days, followed by continuous RAPA or CQ injection until the experimental time points. The RAPA- and CQ-treated groups received the same dosage of RAPA or CQ treatment alone, consecutively for three days followed by saline injection (0.6 ml per rat, i.p.), until the experimental time points. Animals in the CYP, CYP+RAPA, and CYP+CQ groups were euthanized with an over-dose of pentobarbital anesthesia at 4 h, 48 h, or 72 h after CYP injection. Animals in the sham CYP, RAPA, and CQ groups were euthanized using an over-dose of pentobarbital at the 4 h time point for comparison.

### Co-location of microtubule-associated protein 1 light chain 3 (LC3) and alpha-smooth muscle actin (α-SMA) with double-labeled immunofluorescence

The weight of each rat in each of the six groups was recorded prior to sacrifice at each time point. The whole urinary bladder was then procured, weighed, and divided into two parts. The left side of the bladder wall containing the mucosa and urothelium was immediately removed under a dissecting microscope and prepared for western blot. The right side of the bladder wall was divided into three parts, which was used for subsequent study. The right bladder wall of the rats (n = 7) were sectioned with a cryostat microtome (Leica CM1850, Leica Microsystems, Wetzlar, Germany) at 6 μm per slice, and fixed with 4% paraformaldehyde at 4°C for 15 min. The samples were incubated in 0.3% Triton X-100 in PBS for 5 minutes, followed by 1% BSA for 2 hours at room temperature. The specimens were incubated in primary mouse polyclonal anti-α-SMA,1:100 (Santa Cruz Biotechnology, sc-32251),and rabbit polyclonal anti-LC3, 1:300 (sigma, L-7543) antibodies at 4°C overnight followed by the application of secondary fluorescent Alexa-Fluor-488-conjugated goat anti-mouse IgG, 1:100, (Boster Inc, BA-1101) and Alexa-Fluor-555-conjugated donkey anti-rabbit IgG, 1:300, (Invitrogen, A31572) antibodies at room temperature in the dark room for 2 hours. Subsequently, after washing three times with PBS, nuclear labeling was identified by incubating samples in 4',6-diamidino-2-phenylindole for 5 minutes at room temperature. Lastly, the sections were coverslipped using an anti-fading agent. Specimens were observed under a confocal microscope (LSM780NLO, Zeiss, Germany). Ten typical cells with co-localization of LC3 and α-SMA were delineated as regions of interest (ROIs). The LC3 fluorescent spots were numbered with Image J Software. Tissue incubated in PBS containing 1% BSA was used as a negative control.

### Autophagosome detection with transmission electron microscopy (TEM)

The right bladder wall (n = 7) of the CYP, CYP+RAPA and CYP+CQ groups, as well as the sham CYP group, underwent 48 h treatment for autophagosome detection via TEM. As we described previously [23], bladder tissue was harvested and fixed with 3% glutaric dialdehyde and then with 2% perosmic acid. After ethanol gradient dehydration, the specimens were embedded in Epon812 ethoxyline resin. Samples were sectioned at 50 nm per slice and stained with uranyl acetate-lead citrate, followed by morphology assessment via TEM (Philips CM10, Eindhoven, Netherlands). Under 10000-fold magnification, 3–4 fields were randomly selected from each sample for autophagosome quantification.

### Quantitative analysis of LC3, the phosphorylated form of ribosomal protein S6 (p-p70s6k) and superoxide dismutase 2 (SOD2) using western blot

The left bladder wall (n = 7) in each group was used for western blot, as previously reported [24]. The primary antibodies, including rabbit monoclonal anti-phospho-p70S6K, 1:500 (Cell Signaling Technology, #9234), rabbit polyclonal anti-LC3, 1:600 (sigma, L-7543), rabbit polyclonal anti-SOD2, 1:1000 (Santa Cruz Biotechnology, sc-30080) and mouse polyclonal anti-β-actin, 1:1000 (Santa Cruz Biotechnology, sc-47778) were used followed by the application of the secondary antibodies consisting of, horseradish peroxidase-conjugated goat anti-mouse IgG, 1:4000 (Zhongshan Inc, ZB2305) and goat anti-rabbit IgG, 1:1000 (Zhongshan Inc, ZB-2301). The protein band images were collected and the relative optical density (R.O.D) was analyzed with molecular image, ChemiDoc XRS+Image System (Bio-Rad Laboratories, Hercules, CA, USA).

### Histological evaluation

The right bladder wall of rats (n = 7) were sectioned (5μm per slice) for histological analysis. HE and toluidine blue (Shengong Biotech, TE847) staining were applied and subsequently evaluated by an investigator blinded to the treatment groups using a histological score and mast cell counts. In short, histological slides were graded by a score of 0–5 as previous reported [25]. The number of mastocytes in the muscular layer was estimated at 200x magnification in 3 random sections from each group.

### Measurement of IL-1β, IL-6, IL-8, MDA, and GSH with ELISA

Following euthanasia, the mucosa and urothelium were immediately removed from 5 rats in each group, as discussed above for ELISA. The levels of IL-1β (Westtang Inc, F15810), IL-6 (Westtang Inc, F15870), IL-8 (Westtang Inc, F15880), GSH (Westtang Inc, F15488) and MDA (Hushang Inc, HE22220) were measured according to the manufacturer’s assay instructions, and measured at 450 nm with an ultraviolet microplate reader (Thermo Scientific Corporation, Massachusetts, USA).

### Determining detrusor overactivity with bladder cystometry

Cystometry was performed in the CYP, CYP+RAPA, CYP+CQ, and sham CYP groups (5 rats each) 48 hours after CYP or sham CYP injection, similar to the method described previously [23], with urethane anesthesia, subcutaneously, 1.0 mg/kg, (sigma, U2500). A human epidural catheter (2-F internal diameter) was inserted into the bladder to infuse saline at body temperature (37–38°C) at a rate of 8 ml/h. Intravesical pressure was recorded continuously. After stabilization for about 30 minutes, urodynamic parameters, including basal pressure (BP), threshold pressure (TP), maximum pressure (MP), micturition frequency (MF), the intercontraction interval (ICI), and bladder capacity (BC) were evaluated.

### Statistical analysis

Statistical analysis for all outcomes were done using SPSS 16.0 by an investigator blinded to the treatment groups and presented as the mean ± standard deviation. The inflammatory degree data were evaluated using the Kruskal-Wallis test, and the other data were analyzed with one-way ANOVA, followed by the Bonferroni test for multiple comparisons. P <0.05 is considered statistically significant.

## Results

### Change in bladder weight and the ratio of bladder weight to body weight

The bladder weight (Table 1) and ratio of bladder weight to body weight (Table 2) in the CYP, CYP+RAPA, and CYP+CQ groups were significantly enhanced compared to the values in each control group. Values in the CYP+CQ group at each time point were significantly increased compared to those in the CYP group. However, with RAPA treatment, values were significantly decreased compared to the CYP group values.

**Table 1:** Bladder weight (mg) of control CYP, CYP+RAPA and CYP+CQ-treated rats.

Bladder weight	Control	4 h	48 h	72 h
**CYP**	85.56±7.91	212.33±13.95^a^	183. 78±17.23^a^	164. 78±15.58^a^
**CYP+RAPA**	86.44±7.95	161.00±13.94^a^ ^b^	149.11±12.036^a^ ^b^	135.89±11.74^a^ ^b^
**CYP+CQ**	85.00±9.31	242.44±21.51^a^ ^b^	206.11±16.89^a^ ^b^	205.67±13.97^a^ ^b^

Data presented as means ± SD (n = 9).

^a^ indicates a significant difference compared to the corresponding control group value (P <0.05).

^b^ indicates a significant difference compared to the corresponding CYP group value (P <0.05).

**Table 2:** Bladder weight/ body weight (mg/g) of control CYP, CYP+RAPA- and CYP+CQ-treated rats.

Bladder weight/ body weight	Control	4 h	48 h	72 h
**CYP**	0.41±0.04	1.03±0.05^a^	1.03±0.05^a^	0.80±0.07^a^
**CYP+RAPA**	0.40±0.04	0.79±0.07^a^ ^b^	0.74±0.06^a^ ^b^	0.67±0.08^a^ ^b^
**CYP+CQ**	0.40±0.04	1.19±0.10^a^ ^b^	1.07±0.10^a^ ^b^	1.12±0.06^a^ ^b^

Data presented as means ± SD (n = 9).

^a^ indicates a significant difference compared to the corresponding control group value (P <0.05).

^b^ indicates a significant difference compared to the corresponding CYP group value (P <0.05).

### Confirmation of the differences in autophagy in detrusor myocytes with electron microscopy, double-labeled immunofluorescence, and western blot

**Fig 1:**
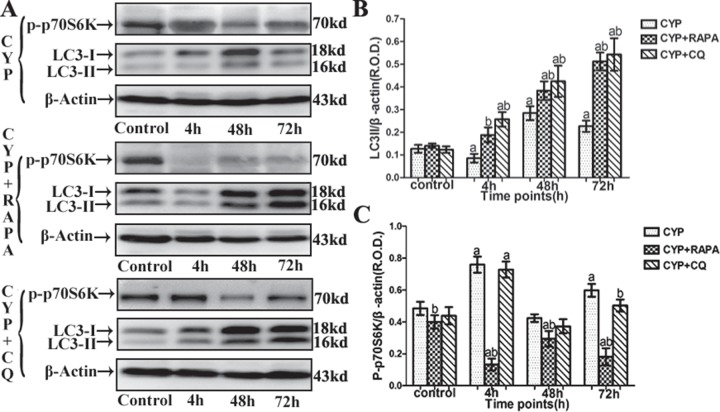
Expression of LC3 and p-p70S6K in bladder muscular layer in CYP-treated rats with Western blot. (A) Comparison of LC3 and phosphorylated (p)-p70S6K expression in the bladder muscular layer of the rats in the CYP-treated, CYP+RAPA-treated, and CYP+CQ-treated groups. β-actin was used as an endogenous control. (B) A statistical chart of relative optical density (R.O.D.) of LC3-II/β-actin in each group, n = 7. (C) A statistical chart of R.O.D. of p-p70S6K/β-actin in the CYP-treated, CYP+RAPA-treated and CYP+CQ-treated groups n = 7. Data are expressed as the mean± SD, ^a^ indicates a significant difference compared to the control group value(P <0.05). ^b^ indicates a significant difference compared to the CYP group value(P <0.05).

**Fig 2:**
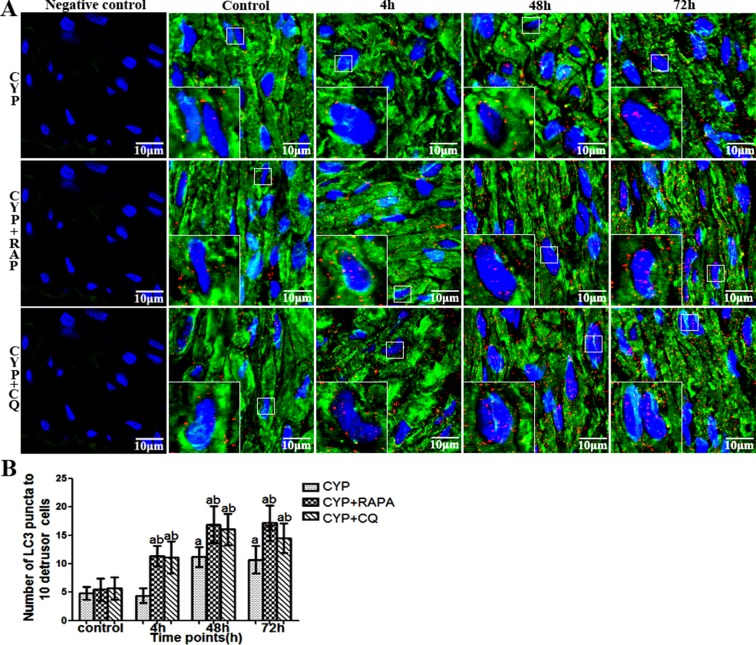
Immunofluorescence co-location of LC3 and α-SMA in bladder muscular layer in CYP-treated rat. (A)Immunofluorescence highlights LC3 (red), α-SMCA (green) and nuclei (blue) in the bladder of rats. (B) Statistical charts show the mean of the numbers of LC3 spots in ten detrusor cells, from rats (n = 7). Data are expressed as the mean ± SD. Scale bars indicate 10 μm in each figure. a indicates a significant difference compared to the control group value(P <0.05). b indicates a significant difference compared to the CYP group value(P <0.05).

**Fig 3:**
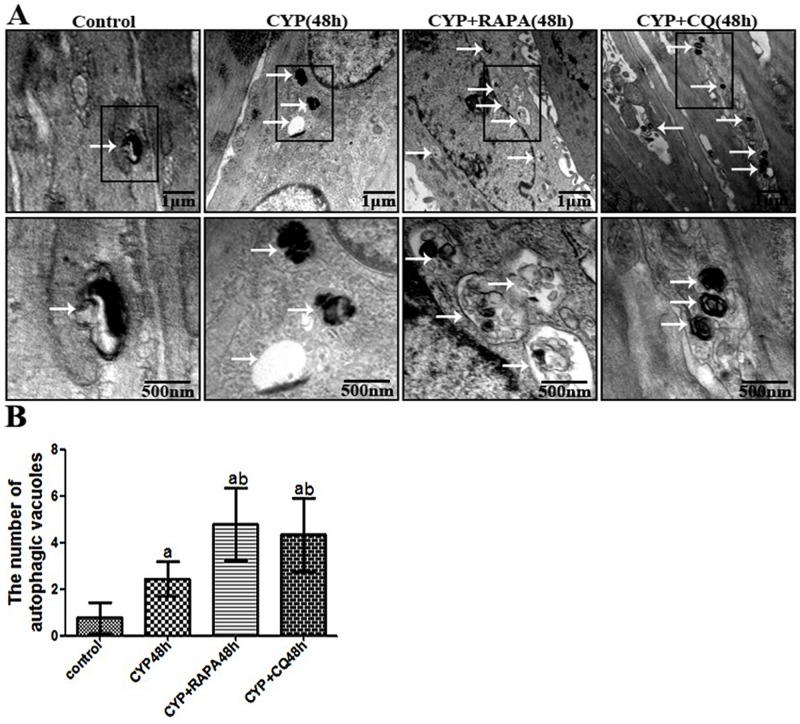
Confirmation of the autophagy in CYP-treated rat detrusor myocytes with electron microscopy. (A) Representative high magnification electron micrographs showing autophagic vacuoles in detrusor myocytes of rat (upper panels: arrows demonstrate vesicles; scale bars, 1 μm) and cytoplasmic goods in autophagic vacuoles (lower panels; scale bars, 500nm), respectively. (B) A statistical chart of the number of autophagic vacuoles in control rats, CYP-treated 48h rats, CYP+RAPA-treated 48h rats, and CYP+CQ-treated 48h rats, n = 7. Data are expressed as the mean ± SD, a indicates a significant difference compared to the control group value(P <0.05). b indicates a significant difference compared to the CYP group value(P <0.05).

Currently, using electron microscopy, immunofluorescence, and western blot with LC-3-II/β-action can detect the change of autophagy [21, 26, 27]. In this tudy, autophagy of detrusor myocytes was confirmed in CYP-induced cystitis with electron microscopy, double-labeled immunofluorescence and LC3 Western blot (Figs 1–3). The number of autophagosomes (Fig 3) detected by electron microscopy in the CYP, CYP+RAPA, and CYP+CQ groups was significantly increased compared to that in the control group. Moreover, significantly higher numbers of autophagosomes were detected in the CYP+RAPA (P <0.05), and CYP+CQ groups (P <0.05) compared to that in the CYP group. Similar results were acquired in LC3 analysis with double-labeled fluorescence and western blot by calibrating agianst the β-actin as internal control (Figs 1 and 2). Moreover, in the CYP+RAPA group, in order to confirmed that RAPA inhibited the mTOR signaling pathway. We detected the expression of p70S6K protein. In this study, the expression of p70S6K in the CYP+RAPA group was significantly decreased compared to that in the CYP and control groups. In the CYP+CQ group, the expression of p70S6K was no significant difference comparing with that of the CYP group (Fig 1).

### Evaluation of inflammatory cytokines and oxidation-related factors using ELISA and western blot

**Fig 4:**
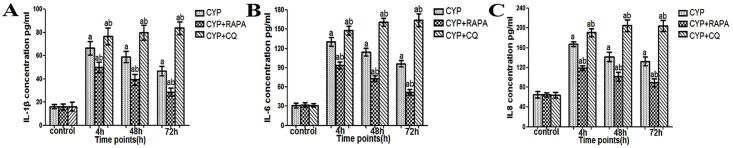
Evaluation of IL-1β, IL-6 and IL-8 changes in the bladder muscular layer in CYP-treated rats using ELISA kits. (A) IL-1β expression, (B) IL-6 expression and (C) IL-8 expression, n = 5. Data are expressed as the mean ± SD, ^a^ indicates a significant difference from normal at each time point (P <0.05). ^b^ indicates a significant difference compared to the CYP group value (P <0.05).

**Fig 5:**
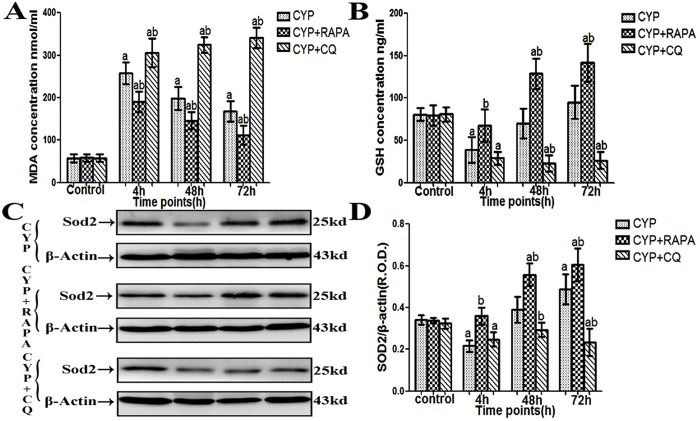
Evaluation of MDA, GSH and SOD2 changes in the bladder muscular layer in CYP-treated rats using ELISA and Western blot. (A) MDA expression. n = 5. (B) GSH expression. n = 5. (C) Representative western blot analysis of bladder muscular layer SOD2 expression in CYP-treated, CYP+RAPA-treated, and CYP+CQ-treated rats. β-actin is used as an endogenous control. (D) A statistical chart of relative optical density (R.O.D.) of SOD2/β-actin in each group, n = 7. Data are expressed as the mean ± SD, ^a^ indicates a significant difference (P <0.05) from normal at each time point. ^b^ indicates a significant difference (P <0.05) from the CYP group values at each time point.

Expression of IL-1β, IL-6, IL-8, and MDA in the bladder muscular layer was significantly increased in the CYP, CYP+RAPA, and CYP+CQ groups at each time point compared to sham CYP, RAPA, and CQ groups, respectively (Figs 4 and 5). Expression of these substances was significantly decreased in the CYP +RAPA group and significantly increased in the CYP+CQ group compared to the CYP group, however. The antioxidant GSH and antioxidant enzyme SOD2 present in the muscular layer was significantly reduced in the CYP group at the 4 h time point but showed an increasing trend at 48 h and 72 h. GSH and SOD2 levels in the CYP +RAPA group were significantly enhanced compared to the CYP group at all time points, and were significantly increased compared to the RAPA group at 48 h and 72 h. In addition, compared to the CQ group, GSH levels in the CYP+CQ group was significantly decreased at all time points. SOD2 was significantly decreased at 4 h and 72 h (Fig 5). Evaluation of both GSH and SOD2 in CYP+CQ group indicated significant differences compared to the CYP group at 48 h and 72 h.

### Histological evaluation

**Fig 6:**
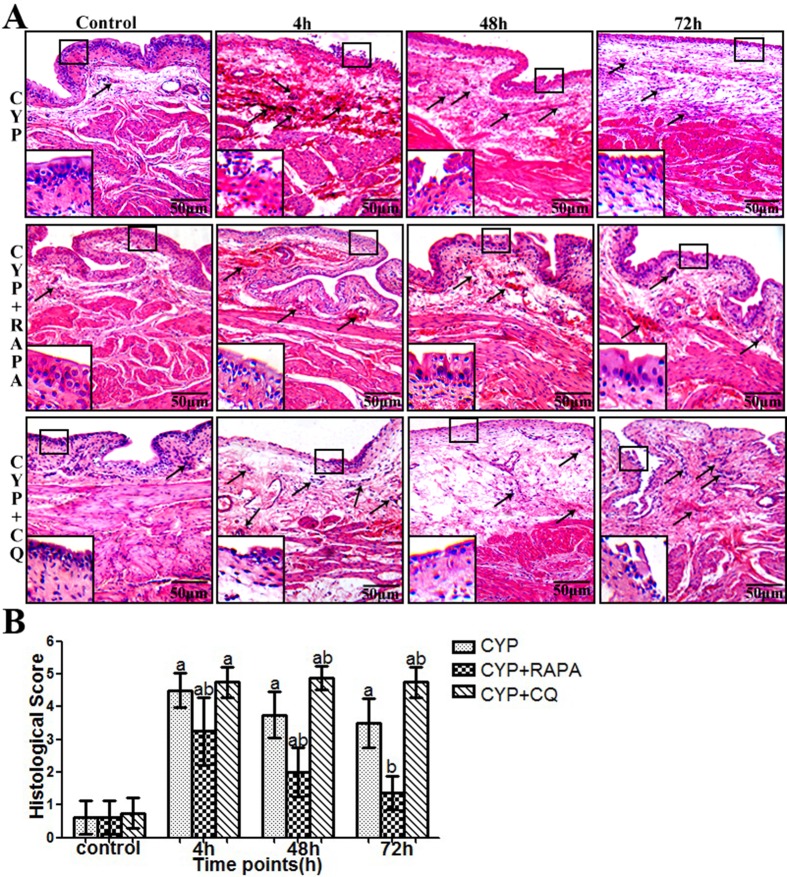
Pathology and inflammation scores in CYP-treated rats using histological evaluation. (A) Representative bladder showing pathologic changes in rat specimens. Staining with hematoxylin and eosin; arrows demonstrate inflammatory cells, (scale bars, 50μm). Inset images show mucosal change. (B) The statistical chart demonstrates the inflammation grading in control, CYP, CYP+RAPA and CYP+CQ treatment, n = 7. Data are expressed as the mean ± SD, ^a^ indicates a significant difference (P ˂0.01) from the control value at each time point. ^b^ indicates a significant difference (P <0.01) from the CYP group value at each time point.

**Fig 7:**
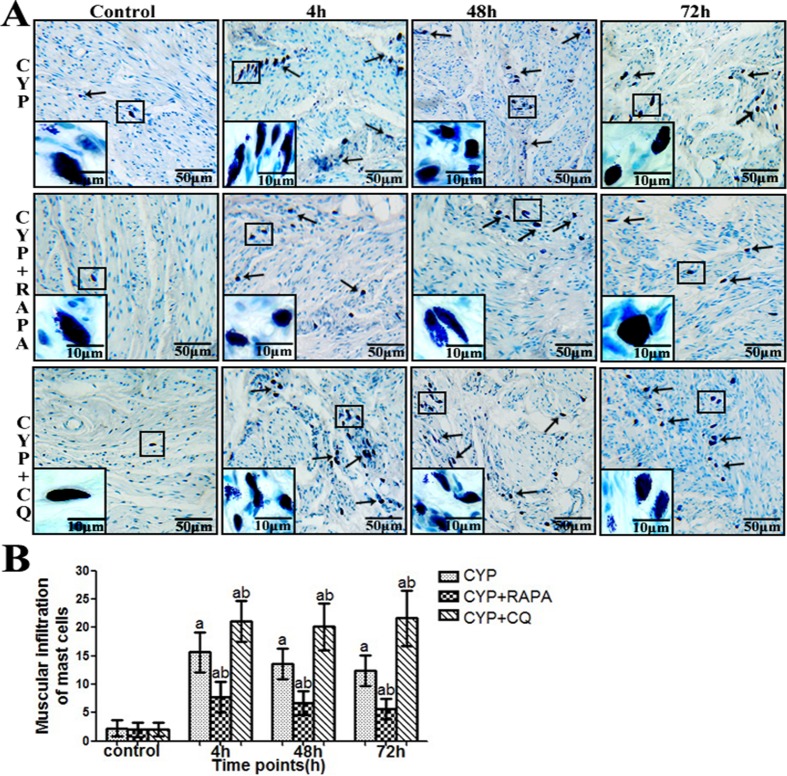
Number of mast cells in CYP-treated rats using histological evaluation. (A) Representative photomicrograph images of the number of mast cells in the muscular layer in rat samples. Staining with toluidine blue (arrows) demonstrate mast cells (scale bars, 50 μm). Inset images show high magnification mast cells (scale bars, 10 μm). (B) A statistical chart reveals the number of mast cells in muscular layer in rats (n = 7). Data are expressed as the mean ± SD; ^a^ indicates a significant difference (P <0.01) from the control group value at each time point. ^b ^indicates a significant difference (P <0.05) from the CYP group value at each time point.

Compared with the control group (Figs 6A and 7A), bladder tissue form CYP-treated rats indicated mssive ulcers, obvious edema and hemorrhage, and increased inflammatory cell infiltration (particularly mast cell) in the submucosal and muscular layer. This trend is more obvious in the CYP+CQ group. However, in the CYP+RAPA group, bladder tissue indicated less mssive ulcers, edema and hemorrhage, and inflammatory cell infiltration (particularly mast cell) in the submucosal and muscular layer. Additionally, the quantitative assessment of histological score and mast cell count (Figs 6B and 7B) demonstrated that the inflammatory response was more severe in the CYP+CQ group, but more varied in the CYP+RAPA group compared to the CYP and control groups.

### Changes in urodynamic parameters

**Fig 8:**
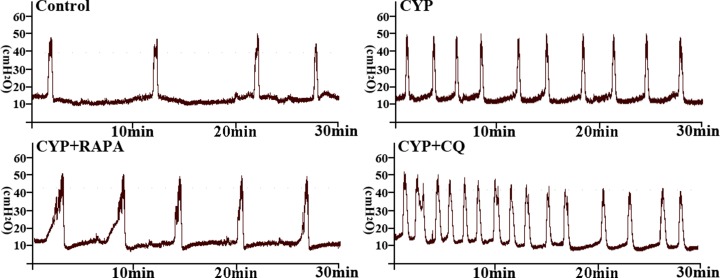
Representative cystometric traces of bladder pressure in sham control, CYP, CYP+RAPA, and CYP+CQ groups.

Compared to the control group (Table 3 and Fig 8), MF was significantly increased, while ICI and BC were decreased in the CYP group at 48 h. However, the bladder function damage trend was more serious in CYP+CQ group at 48 h. In contrast, CYP+RAPA treatment significantly decreased MF but increased ICI and BC compared to the CYP group. No statistical significance was found in these parameters compared to the control group, however, indicating recovered micturition function.

**Table 3:** Cystometric parameters of Control, CYP, CYP+ RAPA and CYP+CQ treated rats.

Urodynamic Parameters	Control	CYP	CYP+RAPA	CYP+CQ
**Basal pressure (cm H**_**2**_ **O)**	10.00±1.37	13.60±2.75	11.30±2.44	14.50±1.97^a^
**Threshold pressure (cm H**_**2**_ **O)**	42.10±3.36	36.70±2.33	37.50±2.50	38.80±3.40
**Maximum pressure (cm H**_**2**_ **O)**	52.90±3.29	51.10±5.57	50.90±5.27	50.90±3.86
**Micturition frequency (No/h)**	7.60±1.14	19.20±2.59^a^	9.60±1.14^b^	28.60±2.61^a^ ^b^
**Intercontraction interval (mins)**	7.95±1.35	3.17±0.42^a^	6.43±0.66^b^	2.11±0.20^a^ ^b^
**Bladder capacity**	1.07±0.17	0.42±0.06^a^	0.89±0.07^b^	0.28±0.036^a^ ^b^

Data presented as means ± SD (n = 5).

^a^ indicates a significant difference compared to the corresponding control group value (P <0.05).

^b^ indicates a significant difference compared to the corresponding CYP group value (P <0.05).

## Discussion

Currently, using electron microscopy, immunofluorescence, and western blot with LC3 as a well-known maker of autophagy [26–28], researchers have detected and measured autophagy activity in the vascular system, respiratory system, and the corpus cavernosum, suggesting its important role in tissue protection [13–15]. In this study, we firstly detected autophagy in detrusor myocytes in rats using electron microscopy, western blot, and double-labeled immunofluorescence. While the autophagy of detrusor myocytes in rats maintain at a low level, it could maintain cellular homeostasis in clearing the aging, misfolded and damaged proteins.

Autophagy is believed to play an important role in cytoprotection and in controlling the inflammatory response by clearing damaged organelles and misfolded proteins [7]. Detrusor myocytes autophagy in bladder cystitis is not well clarified. To further illustrate the importance of detrusor myocytes autophagy as well as its role in inflammation, samples from CYP-induced cystitis animal models were used. In this study, cystitis was successfully induced by CYP administration in the three experimental groups, as evidenced by inflammatory changes such as ulcers, edema, hemorrhage, and inflammatory cell infiltration in the bladder [16, 17, 25]. Moreover, we observed numerous mast cell infiltration in the bladder muscle layer in CYP-treated rats. Traditionally, detrusor myocytes are considered as a passive character in inflammatory diseases, such as BPS/IC. But, numerous clinical bladder biopsy specimens from BPS/IC patients and animal models of bladder inflammation (including CYP-treated cystitis) contain inflammatory changes (especially mast cell infiltration) in the bladder muscular layer itself [16, 18, 29]. Generally, mast cells are rarely present in bladder muscle tissue and do not circulate in their mature form. Instead, local tissue microenvironmental factors activate mast cell proliferation [30]. In fact, when treated with IL-1β or TNF-α, bladder detrusor myocytes can release inflammatory factors, including IL-6, IL-8, MCP-1, and RANTES, which could promote mast cell recruitment, proliferation and maturation [30–32]. Consequently, the detrusor myocytes could cause mast cell migration and activation in the bladder muscle layer. Currently, increasing evidence suggests that smooth muscle cells in the airway, uterus, and blood vessels participate in the inflammatory response [31–33]. Theoretically, when faced with local microenvironment changes, detrusor myocytes might play important roles in the immune response. In this study, we found detrusor myocytes participate in cyclophosphamide-induced cystitis immune and inflammatory responses by autophagy.

In the CYP-induce cystitis animal model, we found that autophagic activity was reduced, but the presence of inflammatory factors (lL-1β, IL-6, IL-8) and oxidative stress markers MDA reached peak levels and nonenzymic antioxidant GSH and antioxidant enzymes SOD2 was reduced at 4 h after CYP injection. However, the enhancement of the autophagic activity at 48 h and 72 h significantly decreased the inflammatory cytokines (lL-1β, IL-6, IL-8) and oxidative stress markers MDA reduced and nonenzymic antioxidant GSH and antioxidant enzymes SOD2 was enhanced. When faced with oxidative stress and inflammation, autophagy can activate which provide to diversify functionally to confront oxidative stress and inflammation [7]. These data seems to indicate that oxidative stress may appear early than autophagy and insufficient activation of detrusor myocyte autophagy resulted to the inflammation and oxidative stress in bladder muscular wall, and the strengthened detrusor myocyte autophagy lead to the reduction of inflammation and oxidative stress in bladder muscular wall. In order to understand the protective effect of detrusor myocyte autophagy in CYP-induced cystitis, we studied the effects of RAPA and CQ treatment. RAPA is a widely used autophagy agonist that inhibits the mammalian target of rapamycin (mTOR), further preventing phosphorylation of P70S6K and other proteins [19, 20]. Autophagy is a cell-dependent lysosomal degradation biological process. In autophagy, double-membrane autophagosomes envelope and sequester intracellular components and then fuse with lysosomes to form autolysosomes, which degrade their contents to regenerate nutrients [7]. Chloroquine is a pharmacological inhibitor of autophagy by acting as a lysosomotropic agent that raises lysosomal pH to suppress the activity of lysosomal acid hydrolases and hence prevent the maturation and lysosomal degradation of autophagosomes. As previously reported, When chloroquine is used, the autophagosome number and its marker LC3 will increase, but the degradation functional of autophagy will be blocked [21, 22]. In this study, we demonstrated that autophagy in the bladder muscular layer was significantly increased at each time point in the CYP+RAPA and CYP+CQ groups compared to that in the control and CYP-treated groups, as evidenced by LC3-II protein expression, LC3 immunofluorescence, and the presence of autophagosomes. These results suggested that RAPA and CQ play the role of activation and blocking autophagy effect, respectively. Moreover, as previously reported, in order to further assess whether RAPA indeed inhibited mTOR signaling pathway, we examined its effect on p70S6K phosphorylation [34, 35]. In this study, the expression of p70S6K in the CYP+RAPA group was significantly decreased compared to that in the CYP and control groups. These results further demonstrated that RAPA inhibited the mTOR signaling pathway and mTOR signaling pathway could involved in cyclophosphamide-induced cystitis.

We also evaluated inflammatory and oxidative stress levels via protein expression and histology. Interestingly, in the CYP and CYP+RAPA groups, the changes in inflammatory factors and oxidative stress in the bladder muscular layer were inversely proportional to the changes in autophagy. In the CYP and CYP+RAPA groups, enhanced autophagy lead to tissue inflammation score, inflammatory factors (lL-1β, IL-6, IL-8) and oxidative stress markers MDA reduced and nonenzymic antioxidant GSH and antioxidant enzymes SOD2 was increased. In addition, interestingly, the bladder muscle layer of mast cell infiltration were also inversely proportional to the changes in autophagy in the CYP and CYP+RAPA groups. These results is mainly due to mast cell chemoattractant (lL-1β, IL-6, IL-8) which mostly come from the reduced detrusor cells. Oppositely, autophagy in the CYP+CQ group increased dramatically and tissue inflammation score, the bladder muscle layer of mast cell infiltration, inflammatory factors and oxidative stress were consistently increased compared to the CYP and control group due to CQ inhibition of autophagic function. These results further demonstrated that blocking the physiological function of autophagy will cause tissue injury, inflammation, oxidative stress increased. Therefore, RAPA would be potentially useful in enhancing autophagic activity and inhibiting inflammation and oxidative stress in cystitis. In urodynamic studies, bladder contraction frequency increased in the CYP and CYP+CQ groups, while voiding intervals shortened and bladder volume decreased. But,This bladder function damage trend is more serious in CYP+CQ group. Recovered bladder function was seen in the CYP+RAPA group, suggesting a better recovery of micturition function due to the enhancement of autophagy. In summary, These results indicates that up-regulation of autophagy will play a protective role in CYP-treated cystitis by reducing inflammation and oxidative stress and thus protecting and improving bladder function.

Although CYP-induced cystitis in rats cannot fully replicate the pathophysiological changes in humans, the CYP-induced cystitis animal model has been commonly used to investigate the pathophysiology of BPS/IC [5, 16–18]. Therefore, our study provides a new perspective and views that detrusor myocyte autophagy could be involved in the pathogenesis of BPS/IC, but withoutspecific regulatory mechanisms of detrusor myocyte autophagy in the pathogeneses of BPS/IC. Further study on the specific molecular mechanisms and human sample is required.

In summary, this study revealed the hidden link between detrusor myocyte autophagy and BPS/IC in rats. The autophagy agonist RAPA significantly decreased inflammation and improved bladder function, and thus could be used as a potential treatment for IC. Further studies are needed to elucidate the regulatory mechanisms of detrusor myocyte autophagy and the effectiveness of RAPA on IC patients.

## Supporting Information

S1 Dataset The original data of the expression of LC3 and p-p70S6K in bladder muscular layer in CYP-treated rats. (XLS)

S2 Dataset The original data of the number of LC3 puncta to 10 detrusor myocytes in CYP-treated rat. (XLS)

S3 Dataset The original data of the number of autophagic vacuoles in CYP-treated rat detrusor myocytes. (XLS)

S4 Dataset The original data of IL-1β, IL-6 and IL-8 changes in the bladder muscular layer in CYP-treated rats. (XLS)

S5 Dataset The original data of MDA, GSH and SOD2 changes in the bladder muscular layer in CYP-treated rats. (XLS)

S6 Dataset The original data of the inflammation grading in CYP-treated rats. (XLS)

S7 Dataset The original data of the number of mast cells in muscular layer in CYP-treated rats. (XLS)

## References

[1] Humphrey L, Arbuckle R, Moldwin R, Nordling J, van de Merwe JP, Meunier J, et al The bladder pain/interstitial cystitis symptom score: development, validation, and identification of a cut score. Eur Urol. 2012;61: 271–279. 10.1016/j.eururo.2011.10.004 22050826

[2] Berry SH, Elliott MN, Suttorp M, Bogart LM, Stoto MA, Eggers P, et al Prevalence of symptoms of bladder pain syndrome/interstitial cystitis among adult females in the United States. J Urol. 2011;186: 540–544. 10.1016/j.juro.2011.03.132 21683389 PMC3513327

[3] Nickel JC, Tripp DA, Pontari M, Moldwin R, Mayer R, Carr LK, et al Psychosocial phenotyping in women with interstitial cystitis/painful bladder syndrome: a case control study. J Urol. 2010;183: 167–172. 10.1016/j.juro.2009.08.133 19913812

[4] Dasgupta J, Tincello DG. Interstitial cystitis/bladder pain syndrome: an update. Maturitas. 2009; 64: 212–217. 10.1016/j.maturitas.2009.09.016 19837525

[5] Nasrin S, Masuda E, Kugaya H, Ito Y, Yamada S. Improvement by phytotherapeutic agent of detrusor overactivity, down-regulation of pharmacological receptors and urinary cytokines in rats with cyclophosphamide induced cystitis. J Urol. 2013;189: 1123–1129. 10.1016/j.juro.2012.09.054 23000860

[6] Heng SJ, Tzu LH, Chorng KH. Protein expression profiling in interstitial cystitis/painful bladder syndrome: A pilot study of proteins associated with inflammation, apoptosis, and angiogenesis. Urological Science. 2012;23: 107–113.

[7] Levine B, Mizushima N, Virgin HW. Autophagy in immunity and inflammation. Nature. 2011; 469: 323–335. 10.1038/nature09782 21248839 PMC3131688

[8] Jo EK, Shin DM, Choi AM. Autophagy: cellular defense to excessive inflammation. Microbes Infect. 2012;14: 119–125. 10.1016/j.micinf.2011.08.014 21924374

[9] Kaser A, Blumberg RS. Endoplasmic reticulum stress in the intestinal epithelium and inflammatory bowel disease. Semin Immunol. 2009;21: 156–163. 10.1016/j.smim.2009.01.001 19237300 PMC4736746

[10] Moscat J, Diaz-Meco MT. p62 at the crossroads of autophagy, apoptosis, and cancer. Cell. 2009;137: 1001–1004. 10.1016/j.cell.2009.05.023 19524504 PMC3971861

[11] Saitoh T, Fujita N, Jang MH, Uematsu S, Yang BG, Satoh T, et al Loss of the autophagy protein Atg16L1 enhances endotoxin-induced IL-1beta production. Nature. 2008;456: 264–268. 10.1038/nature07383 18849965

[12] Tal MC, Sasai M, Lee HK, Yordy B, Shadel GS, Iwasaki A, et al Absence of autophagy results in reactive oxygen species-dependent amplification of RLR signaling. Proc Natl Acad Sci U S A. 2009;106: 2770–2775. 10.1073/pnas.0807694106 19196953 PMC2650341

[13] He C, Zhu H, Zhang W, Okon I, Wang Q, Li H, et al 7-Ketocholesterol induces autophagy in vascular smooth muscle cells through Nox4 and Atg4B. Am J Pathol. 2013;183: 626–637. 10.1016/j.ajpath.2013.04.028 23770348 PMC3730774

[14] Ghavami S, Mutawe MM, Schaafsma D, Yeganeh B, Unruh H, Klonisch T, et al Geranylgeranyl transferase 1 modulates autophagy and apoptosis in human airway smooth muscle. Am J Physiol Lung Cell Mol Physiol. 2012;302: L420–428. 10.1152/ajplung.00312.2011 22160308

[15] Zhang MG, Wang XJ, Shen ZJ, Gao PJ. Long-term oral administration of 5alpha-reductase inhibitor attenuates erectile function by inhibiting autophagy and promoting apoptosis of smooth muscle cells in corpus cavernosum of aged rats. Urology. 2013;82: 743 e749–715. 10.1016/j.urology.2013.02.045 23876578

[16] Chuang YC, Yoshimura N, Huang CC, Wu M, Chiang PH, Chancellor MB. Intravesical botulinum toxin A administration inhibits COX-2 and EP4 expression and suppresses bladder hyperactivity in cyclophosphamide-induced cystitis in rats. Eur Urol. 2009;56: 159–166. 10.1016/j.eururo.2008.05.007 18514386

[17] Juszczak K, Gil K, Wyczolkowski M, Thor PJ. Functional, histological structure and mastocytes alterations in rat urinary bladders following acute and [corrected] chronic cyclophosphamide treatment. J Physiol Pharmacol. 2010;61: 477–482. 20814076

[18] Girard BM, Cheppudira BP, Malley SE, Schutz KC, May V, Vizzard MA. Increased expression of interleukin-6 family members and receptors in urinary bladder with cyclophosphamide-induced bladder inflammation in female rats. Front Neurosci. 2011;5: 20 10.3389/fnins.2011.00020 21373362 PMC3044559

[19] Carames B, Hasegawa A, Taniguchi N, Miyaki S, Blanco FJ, Lotz M. Autophagy activation by rapamycin reduces severity of experimental osteoarthritis. Ann Rheum Dis. 2012;71: 575–581. 10.1136/annrheumdis-2011-200557 22084394 PMC3294168

[20] Erlich S, Alexandrovich A, Shohami E, Pinkas-Kramarski R. Rapamycin is a neuroprotective treatment for traumatic brain injury. Neurobiol Dis. 2007;26: 86–93. 17270455 10.1016/j.nbd.2006.12.003

[21] Jiang M, Liu K, Luo J, Dong Z. Autophagy is a renoprotective mechanism during in vitro hypoxia and in vivo ischemia-reperfusion injury. Am J Pathol. 2010;176: 1181–1192. 10.2353/ajpath.2010.090594 20075199 PMC2832141

[22] Amaravadi RK, Yu D, Lum JJ, Bui T, Christophorou MA, Evan GI, et al Autophagy inhibition enhances therapy-induced apoptosis in a Myc-induced model of lymphoma. J Clin Invest. 2007;117: 326–336. 17235397 10.1172/JCI28833 PMC1765515

[23] Li L, Jiang C, Hao P, Li W, Song C, Song B. Changes of gap junctional cell-cell communication in overactive detrusor in rats. Am J Physiol Cell Physiol. 2007;293: C1627–1635. 17855776 10.1152/ajpcell.00122.2007

[24] Chen W, Jiang C, Jin X, Shen W, Song B, Li L. Roles of stem cell factor on loss of interstitial cells of Cajal in bladder of diabetic rats. Urology. 2011;78: 1443 e1441–1446. 10.1016/j.urology.2011.08.019 22000930

[25] Starkman JS, Martinez-Ferrer M, Iturregui JM, Uwamariya C, Dmochowski RR, Bhowick NA. Nicotinic signaling ameliorates acute bladder inflammation induced by protamine sulfate or cyclophosphamide. J Urol. 2008;179: 2440–2446. 10.1016/j.juro.2008.01.082 18433785

[26] Klionsky DJ, Abdalla FC, Abeliovich H, Abraham RT, Acevedo-Arozena A, Adeli K, et al Guidelines for the use and interpretation of assays for monitoring autophagy. Autophagy. 2012;8:445–544. 22966490 10.4161/auto.19496 PMC3404883

[27] Galluzzi L, Aaronson SA, Abrams J, Alnemri ES, Andrew DW, Baehrecke EH, et al Guidelines for the use and interpretation of assays for monitoring cell death in higher eukaryotes. Cell Death Differ. 2009;16:1093–107. 10.1038/cdd.2009.44 19373242 PMC2757140

[28] Mangieri LR, Mader BJ, Thomas CE, Taylor CA, Luker AM, Tse TE, et al ATP6V0C knockdown in neuroblastoma cells alters autophagy-lysosome pathway function and metabolism of proteins that accumulate in neurodegenerative disease. PLoS One. 2014;9:e93257 10.1371/journal.pone.0093257 24695574 PMC3973706

[29] Sant GR, Kempuraj D, Marchand JE, Theoharides TC. The mast cell in interstitial cystitis: role in pathophysiology and pathogenesis. Urology. 2007;69: 34–40. 17462477 10.1016/j.urology.2006.08.1109

[30] Galli SJ, Nakae S, Tsai M. Mast cells in the development of adaptive immune responses. Nat Immunol. 2005; 6: 135–142. 15662442 10.1038/ni1158

[31] Bouchelouche K, Andresen L, Alvarez S, Nordling J, Nielsen OH, Bouchelouche P, et al Interleukin-4 and 13 induce the expression and release of monocyte chemoattractant protein 1, interleukin-6 and stem cell factor from human detrusor smooth muscle cells: synergy with interleukin-1beta and tumor necrosis factor-alpha. J Urol. 2006;175: 760–765. 16407046 10.1016/S0022-5347(05)00167-9

[32] Bouchelouche K, Alvarez S, Horn T, Nordling J, Bouchelouche P. Human detrusor smooth muscle cells release interleukin-6, interleukin-8, and RANTES in response to proinflammatory cytokines interleukin-1beta and tumor necrosis factor-alpha. Urology. 2006;67: 214–219. 16413378 10.1016/j.urology.2005.07.049

[33] Helmer H, Tretzmuller U, Brunbauer M, Kaider A, Husslein P, Knofler M. Production of oxytocin receptor and cytokines in primary uterine smooth muscle cells cultivated under inflammatory conditions. J Soc Gynecol Investig. 2002; 9: 15–21. 11839503 10.1016/s1071-5576(01)00142-3

[34] Erlich S, Alexandrovich A, Shohami E, Pinkas-Kramarski R. Rapamycin is a neuroprotective treatment for traumatic brain injury. Neurobiol Dis. 2007;26:86–93. 17270455 10.1016/j.nbd.2006.12.003

[35] Pan T, Kondo S, Zhu W, Xie W, Jankovic J, Le W. Neuroprotection of rapamycin in lactacystin-induced neurodegeneration via autophagy enhancement. Neurobiol Dis. 2008;32:16–25. 10.1016/j.nbd.2008.06.003 18640276

